# Different Diffusion Models in the Diagnosis of Brain Microstructural Changes in Post-stroke Depression Patients: A Comparative Study

**DOI:** 10.7759/cureus.82476

**Published:** 2025-04-17

**Authors:** Fang Zhang, Jing Zhang, Lei Zhang, Hengjun Jin, Daqing Li, Wei Zhao

**Affiliations:** 1 Radiology, Huaibei People's Hospital, Huai Bei, CHN

**Keywords:** brain microstructural change, diagnostic efficacy, diffusion models, gray matter, post-stroke depression

## Abstract

Objective

The study aimed to compare the diagnostic effectiveness of different diffusion models in patients with post-stroke depression (PSD) by examining associated gray matter microstructural changes.

Methods

Twenty-nine acute cerebral infarction patients (10 with PSD (mean age: 55.20±8.64 years, four female), 19 without PSD (mean age: 63.26±8.69 years, eight female)), and 18 age- and gender-matched healthy people (mean age: 58.67±9.02 years, eight female) were included. Their age, gender, body mass index, education level, insomnia status, and cognitive assessment scores were analyzed. All underwent diffusion spectrum imaging scans. Three diffusion models (diffusion kurtosis imaging (DKI), diffusion tensor imaging (DTI), and neurite orientation dispersion and density imaging (NODDI)) were used to obtain parameters. Whole-brain analysis was done to find gray matter regions with PSD-related changes and evaluate model efficacy.

Results

PSD patients had higher depression scores and lower average education levels. Regions like the middle frontal gyrus were studied. In the control and PSD group comparison, NODDI_ODI_p10 in the precuneus had the highest diagnostic efficacy, with an area under the curve (AUC) of 0.817. For the control and non-PSD groups, DKI_MD_p10 in the anterior cingulate gyrus was most effective, AUC = 0.898. In the PSD and non-PSD group comparison, DTI_MD_p25 in the amygdala had the highest efficacy, AUC = 0.816.

Conclusion

PSD patients show microstructural abnormalities. Diffusion models can help detect early PSD-related damage, with the neurite orientation dispersion and density imaging model showing promise. However, different models have different efficacies in various group comparisons, and more large-scale research is needed to confirm their diagnostic value.

## Introduction

Stroke currently ranks as the primary health threat among Chinese residents, characterized by high incidence, mortality, and disability rates. Post-stroke depression (PSD), a prevalent complication of stroke, maintains a consistently high overall incidence [1,2,3]. PSD not only exacerbates patients' psychological burden but also impairs their neurological function, leading to increased disability and mortality rates and severely compromising their quality of life and prognosis.

Patients with PSD often present symptoms such as mood fluctuations, sluggish movement, irritability or apathy, anhedonia, pessimism, and suicidal ideation. Approximately one-third of stroke survivors are afflicted by PSD, and it can manifest at any stage following a stroke [4]. Compared to non-depressed patients, PSD patients typically have a poorer prognosis, lower quality of life, a higher recurrence rate of vascular events, and a higher mortality rate [5]. Therefore, identifying the risk factors associated with PSD is of utmost importance. Numerous factors, including insomnia, serum homocysteine levels, and stroke severity, may be linked to PSD [6,7].

As a distinct form of depression, the theory of PSD-related emotional circuit damage has received extensive research recently. The limbic-cortical-striatal-pallidal-thalamic (LCSPT) circuit is closely associated with negative emotions [8]. The normal function of this circuit depends on the integrity of brain fibers. When a patient experiences a stroke, it may directly or indirectly disrupt the neural circuits involved in emotion control. Abnormalities in gray matter structure may be the underlying cause of neuronal network dysfunction [9].

Currently, diffusion tensor imaging (DTI) is one of the primary imaging techniques for studying gray matter integrity. This technique is based on the normal tissue standard distribution of water molecules in each tissue [10]. However, in the human body, diffusion barriers such as cell membranes restrict the diffusion of water molecules in tissues, causing them to deviate from a Gaussian distribution. As an extension of DTI, the diffusion kurtosis imaging (DKI) employed in this study is a method for exploring the non-Gaussian distribution diffusion characteristics of water molecules. It can overcome the limitations of DTI, observe the changes in brain microstructures under multiple b-values, describe the diffusion signal more precisely, and provide more abundant information regarding tissue microstructures [11,12].

Neurite orientation dispersion and density imaging (NODDI) is also a significant technique. As a relatively novel magnetic resonance imaging technique, NODDI can quantitatively analyze the orientation dispersion and density information of neurites in brain tissue at the micro-level. By using NODDI, parameters such as the neurite orientation dispersion index (ODI), intracellular volume fraction (ICVF), and extracellular volume fraction (ECVF) can be obtained. These parameters assist in revealing the characteristics and changes of the micro-structure of neural tissues [13,14]. Thus, in this study, three diffusion models, namely the diffusion kurtosis model (DKI), the diffusion tensor model (DTI), and the neurite orientation dispersion and density model (NODDI), were compared to explore the diagnostic efficacy of the quantitative parameters of different models for PSD.

## Materials and methods

Research subjects

This study included 29 post-stroke patients. All data were sourced from acute cerebral infarction patients hospitalized in our hospital from March 1, 2023, to February 29, 2024. The inclusion criteria were as follows: all subjects were diagnosed by cranial CT or MRI and had experienced their first-onset stroke. The exclusion criteria were as follows: 1. Patients with communication difficulties, hearing impairments, or other severe cognitive impairments that precluded cooperation during the examination; 2. Patients with other cerebrovascular diseases, such as cerebral hemorrhage and subarachnoid hemorrhage; 3. Patients with a history of depression or mental disorders who were taking or had taken antidepressant medications; and 4. Patients with metal substances in their bodies or those with contraindications to magnetic resonance imaging.

This study was approved by the ethics committee of Huaibei People's Hospital, Huai Bei, China (approval number 2023-012). All participants were right-handed, married, and provided written informed consent. Two weeks after admission, patients' cognitive function and mental health status were evaluated using the Mini-Mental State Examination (MMSE), Montreal Cognitive Assessment (MoCA), Activities of Daily Living Scale (ADL), and Hamilton Depression Scale-21 items (HAMD-21). Based on the scale scores, patients were divided into two groups: post-stroke depression (PSD) and post-stroke non-depression (Non-PSD).

Ultimately, 19 non-depressed stroke patients (Non-PSD group, eight females, age: 63.26±8.69 years), 10 PSD patients (four females, age: 55.20±8.64 years), and 18 age-and gender-matched healthy controls (eight females, age: 58.67±9.02 years) were enrolled.

MRI scanning

A MAGNETOM VIDA 3T MRI scanner (Siemens Healthineers, Erlangen, Germany) was used for MRI scanning two weeks after admission. The scanning sequences encompassed T1-weighted images, T2-weighted images, and a diffusion spectrum imaging (DSI) sequence (Table 1).

**Table 1:** Imaging parameters TR: repetition time; TE: echo time; FOV: field of view; MPRAGE: Magnetization-Prepared Rapid Gradient Echo; sag/tra: saggital/transverse; DSI: diffusion spectrum imaging

	T1 MPRAGE	T2 sag/tra	DSI
TR (ms)	2200	4000	2900
TE (ms)	2.46	107	116
FOV (mm×mm)	230×230	240×240	220×220
Resolution (mm³)	0.9×0.9×0.9	0.6×0.6×5	0.8×0.8×2.2
Slice	208	18	60
Scan time	5 min 21 s	45 s	6 min 54 s

T1 images were acquired using 3D SPACE (Sampling Perfection with Application optimized Contrasts using different flip angle Evolutions)** **and utilized for brain segmentation. The specific parameters were as follows: repetition time (TR) 2200 ms, echo time (TE) 2.46 ms, field of view (FOV) 230 mm, resolution 0.9×0.9 mm², slice thickness 0.9 mm, number of slices 208, and scanning time 5 minutes and 21 seconds.

T2 images were scanned in the transverse and sagittal planes. The specific parameters were as follows: TR 4000 ms, TE 107 ms, FOV 240 mm, resolution 0.6×0.6 mm², slice thickness 5 mm, number of slices 18, and scanning time 45 seconds.

The DSI scan was conducted in the transverse plane. The specific parameters were as follows: TR 2900 ms, TE 116 ms, FOV 22 cm, resolution 0.8×0.8 mm², number of slices 60, slice thickness 2.2 mm, acquisition mode q-space, maximum b-value 3000 s/mm², 128 diffusion-sensitive directions, and scanning time 6 minutes and 54 seconds.

Data post-processing and analysis

**Figure 1:**
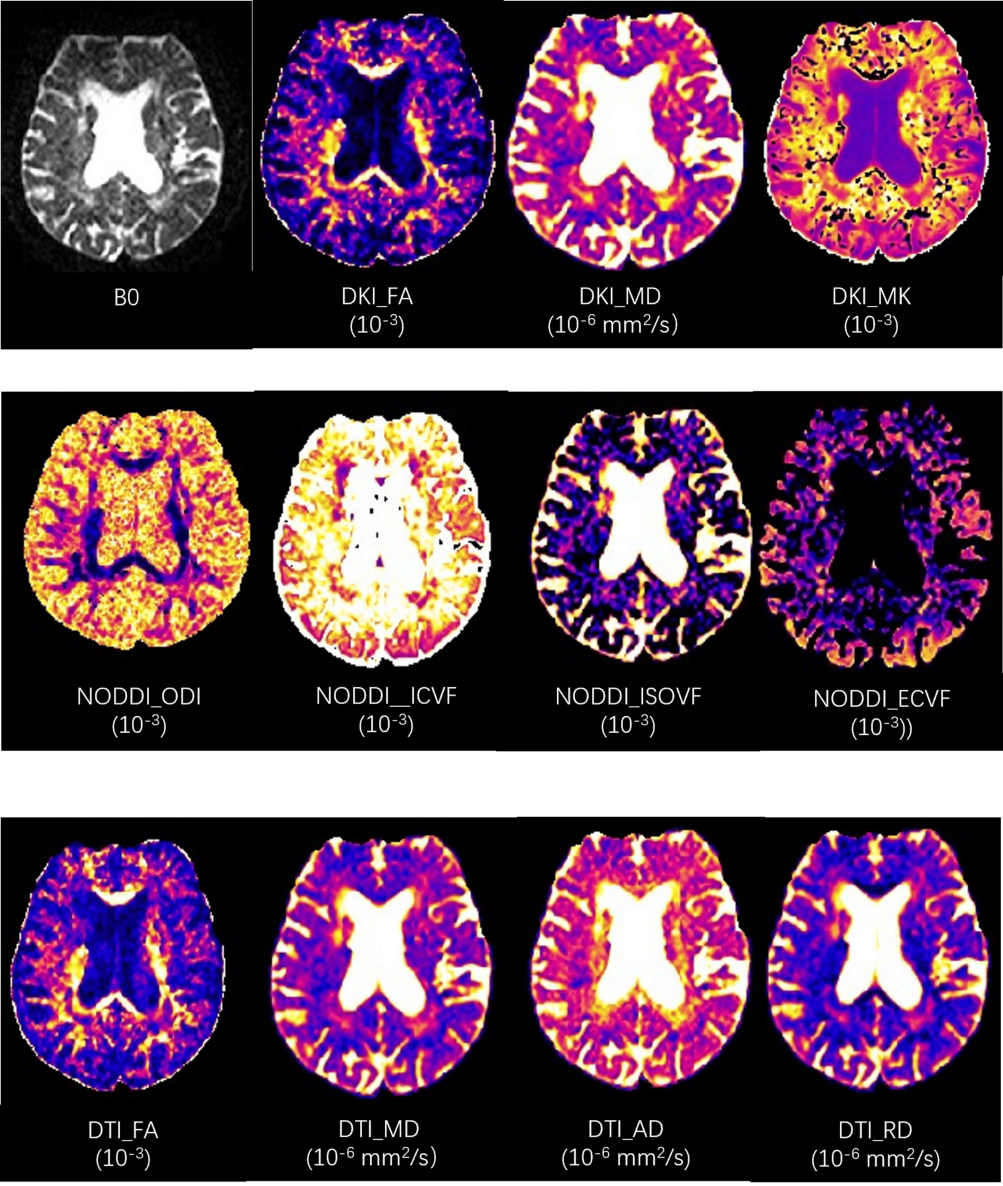
Images of diffusion parameters A 67-year-old male patient was treated in our hospital after a stroke. After two weeks of discharge, scale scores and MRI imaging were performed to evaluate post-infarction depression. The figure shows B0 and the parameters obtained by three diffusion models. The first behavior is the quantitative parameters of the diffusion kurtosis imaging (DKI) model, the second behavior is the quantitative parameters of the neurite orientation dispersion and density imaging (NODDI) model, and the third behavior is the quantitative parameters of the diffusion tensor imaging (DTI) model. The units of each parameter are in parentheses. DKI_FA: diffusion kurtosis imaging fractional anisotropy; DKI_MD: kurtosis imaging mean diffusivity; DKI_MK: Kurtosis imaging mean kurtosis; NODDI_ODI: neurite orientation dispersion and density imaging neurite orientation dispersion index; NODDI_ICVF: neurite orientation dispersion and density imaging intracellular volume fraction; NODDI_ECVF: neurite orientation dispersion and density imaging extracellular volume fraction; NODDI_ISOVF: neurite orientation dispersion and density imaging isotropic volume fraction; DTI_FA: diffusion tensor imaging fractional anisotropy; DTI_MD: diffusion tensor imaging mean diffusivity; DTI_AD: diffusion tensor imaging axial diffusivity; DTI_RD: diffusion tensor imaging radial diffusivity

All diffusion-quantitative parameters were analyzed using NeuDiLab software (Chengdu Zhongying, Chengdu, China). The obtained quantitative parameters included diffusion kurtosis imaging diffusion fractional anisotropy(DKI_FA), kurtosis imaging mean diffusivity (DKI_MD), diffusion kurtosis imaging mean kurtosis (DKI_MK), diffusion tensor imaging axial diffusivity (DTI_AD), diffusion tensor imaging fractional anisotropy (DTI_FA), diffusion tensor imaging mean diffusivity (DTI_MD), diffusion tensor imaging radial diffusivity (DTI_RD), neurite orientation dispersion and density imaging extracellular volume fraction (NODDI_ECVF), neurite orientation dispersion and density imaging intracellular volume fraction (NODDI_ICVF), neurite orientation dispersion and density imaging isotropic volume fraction (NODDI_ISOVF), and neurite orientation dispersion and density imaging neurite orientation dispersion index (NODDI_ODI) (Figure 1).

The quantitative parameters of all patients were registered to the Montreal Neurological Institute (MNI) standard-space template based on the SPACE structural-image data and segmented using the anatomical automatic labeling​​​​​​​ (AAL) template. All operations were completed with statistical parametric mapping​​​​​​​ (SPM). 116 brain regions and 10 diffusion-quantitative parameters (two for DKI, two for DTI, and six for NODDI) of the corresponding brain regions were obtained, and the 10th, 25th, 50th, 75th, and 90th percentiles were extracted. All the extracted parameters were compared among the three groups using the Kruskal-Wallis test to finally select the brain regions and quantitative-parameter features with significant differences. Subsequently, the receiver operating characteristic (ROC) curve analysis was performed on the selected features among different groups to evaluate the diagnostic efficacy. All statistical analyses were conducted using SPSS 19.0 software (IBM Corp., Armonk, USA). A P-value less than 0.05 was considered statistically significant.

For basic patient information such as age, gender, BMI, education level, insomnia status, and cognitive assessment scales, if the data conformed to a normal distribution, analysis of variance (ANOVA) was used for statistics; if not, non-parametric analysis was employed. A P-value less than 0.05 was considered to indicate a statistical difference.

## Results

A statistical analysis of basic information in the normal, non-post-stroke depression (Non-PSD), and post-stroke depression (PSD) groups revealed differences (Table 2).

**Table 2:** Patients’ and healthy volunteers’ general information * p<0.05 ** *p*<0.01 MoCA: Montreal Cognitive Assessment; HAMD: Hamilton Depression Scale; ADL: Activities of Daily Living; MMSE: Mini-Mental State Examination; Non-PSD: non-post-stroke depression group; PSD: post-stroke depression group

	Type			
	Normal	Non_PSD	PSD	p
Age	58.67±9.02	63.26±8.69	55.20±8.64	0.061
Sex	2.000(1.0,2.0)	2.000(1.0,2.0)	2.000(1.0,2.0)	0.974
BMI	24.40±2.68	25.54±3.02	25.24±2.81	0.471
Level of education	9.06±5.13	5.37±4.65	7.80±2.10	0.049*
Insomnia	1.000(1.0,1.0)	1.000(1.0,2.0)	1.000(1.0,2.0)	0.299
MoCA	/	15.37±6.15	15.90±5.49	0.820
HAMD	/	7.42±2.39	15.20±3.74	<0.001**
ADL	/	100.000(70.0,100.0)	100.000(100.0,100.0)	0.037*
MMSE	/	20.000(15.0,23.0)	21.000(17.5,27.0)	0.382

In terms of education level, the Non-PSD group had a significantly lower mean level of education (5.37±4.65) compared to the normal (9.06±5.13) and PSD (7.80±2.10) groups. There were no significant differences in age, BMI, or gender.

Regarding cognitive assessment scales, HAMD and ADL scores showed significant differences between the PSD and Non-PSD groups, indicating poorer daily living ability in PSD patients.

For patients with PSD, the areas of cerebral infarction are mainly concentrated in the corona radiata, basal ganglia region, thalamus, occipital lobe, brainstem, and temporal lobe. For Non-PSD patients, the areas of cerebral infarction are mainly concentrated in the basal ganglia region, corona radiata, temporal lobe, frontal lobe, parietal lobe, brainstem, and corpus callosum.

Figure 1 presents the quantitative parameter maps of DKI, DTI, and NODDI for patients. Through statistical analysis among the three groups, the quantitative parameters of gray matter regions such as the middle frontal gyrus, superior medial frontal gyrus, anterior cingulate gyrus, paracingulate gyrus, posterior cingulate gyrus, amygdala, and precuneus demonstrated significant differences. Consequently, these regions were selected as the regions of interest.

Diagnostic efficacy

**Figure 2:**
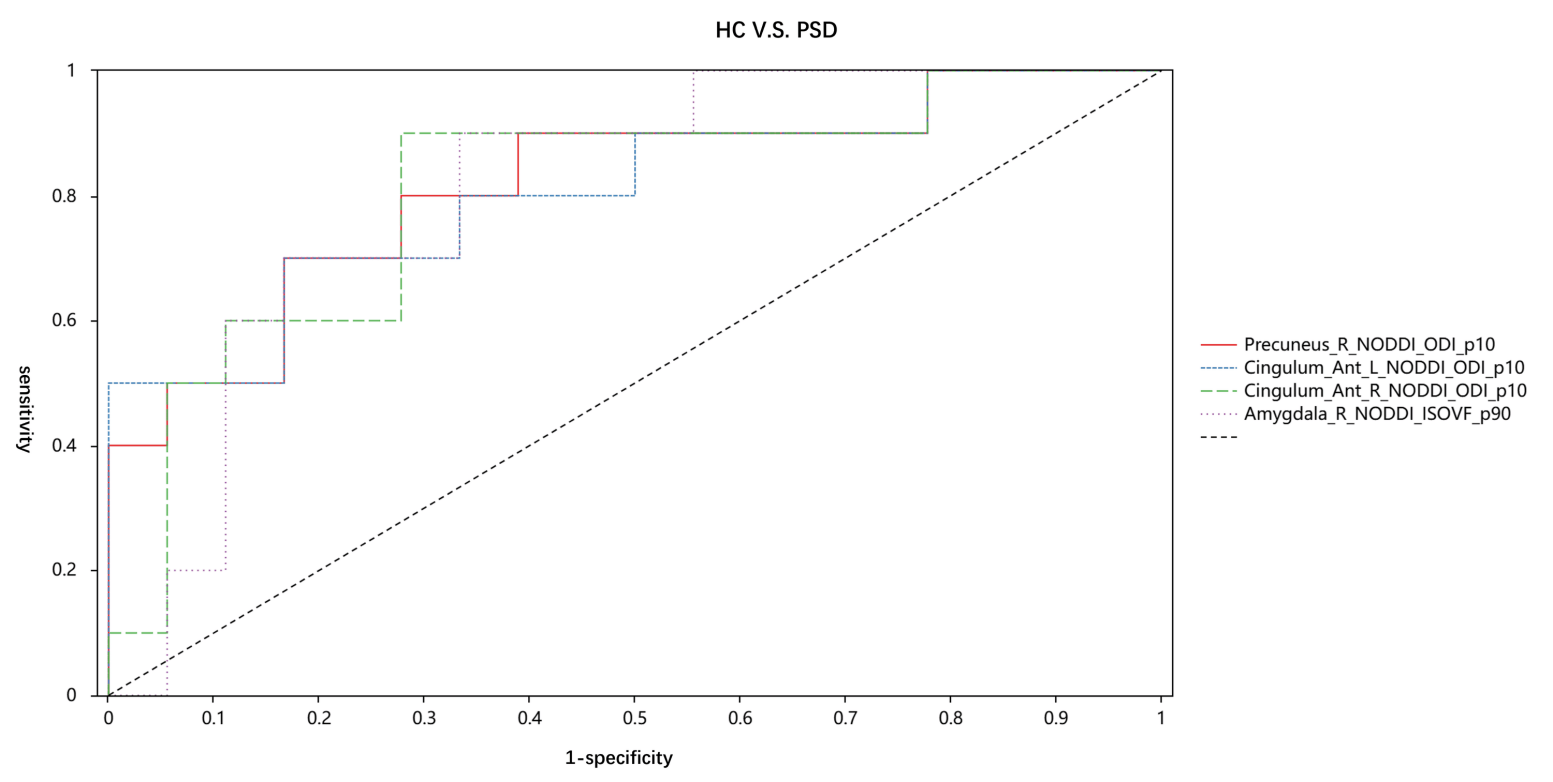
Areas under the receiver operating characteristic curve (AUCs) of healthy controls (HC) with post-stroke depression (PSD) patients The AUC of Precuneus_R_NODDI_ODI_p10 is 0.817. The AUC of Cingulum_Ant_L_NODDI_ODI_p10 is 0.806. The AUC of Cingulum_Ant_R_NODDI_ODI_p10 is 0.806. The AUC of Amygdala_R_NODDI_ISOVF_p90 is 0.806.

**Figure 3:**
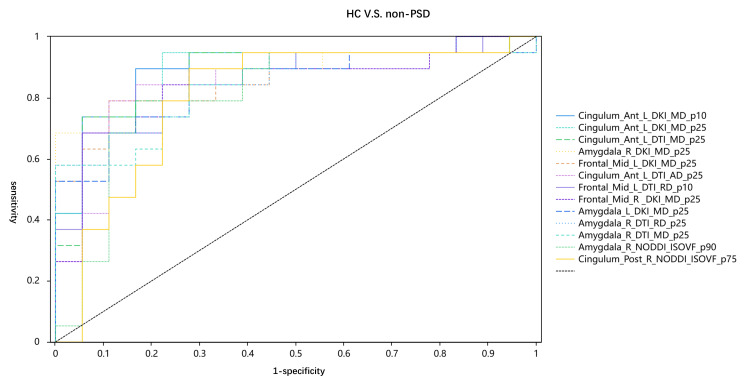
Areas under the receiver operating characteristic curve (AUCs) of healthy controls (HC) with non-post-stroke deptression (non-PSD) patients The AUC of Cingulum_Ant_L_DKI_MD_p10 is 0.898. The AUC of Cingulum_Ant_L_DKI_MD_p25 is 0.886. The AUC of Cingulum_Ant_L_DTI_MD_p25 is 0.874. The AUC of Amygdala_R_DKI_MD_p25 is 0.865.  The AUC of Frontal_Mid_L_DKI_MD_p25 is 0.857.  The AUC of Cingulum_Ant_L_DTI_AD_p25 is 0.854.  The AUC of Frontal_Mid_R _DKI_MD_p25 is 0.851.  The AUC of Frontal_Mid_R _DKI_MD_p25 is 0.845.  The AUC of Amygdala_L_DKI_MD_p25 is 0.845.  The AUC of Amygdala_L_DKI_MD_p25 is 0.845.  The AUC of Amygdala_L_DKI_MD_p25 is 0.842.  The AUC of Amygdala_R_NODDI_ISOVF_p90 is 0.81.  The AUC of Amygdala_R_DKI_MD_p25 is 0.804.

**Figure 4:**
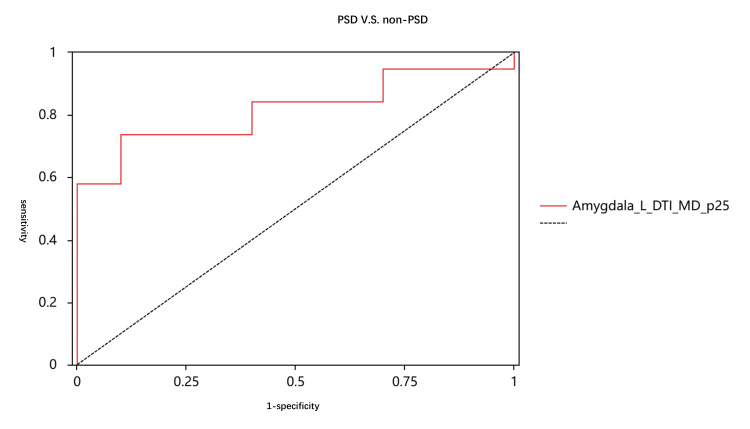
Areas under the receiver operating characteristic curve (AUCs) of post-stroke depression (PSD) with non-PSD patients The AUC of Amygdala_L_DTI_MD_p25 is 0.816.

Figures 2-4 and Tables 3-5 display the diagnostic performance results of the quantitative parameters with significant differences among the three groups. In the comparison between the PSD group and the normal group, the NODDI_ODI_p10 of the precuneus had the highest diagnostic efficacy, with an area under the curve (AUC) of 0.817. The diagnostic efficacies of NODDI_ODI_p10 in the anterior cingulate gyrus and NODDI_ISOVF_p90 in the amygdala were also higher than 0.8. In the comparison between the Non-PSD group and the normal group, the DKI_MD_p10 of the anterior cingulate gyrus had the highest diagnostic efficacy, with an AUC of 0.898. Additionally, the diagnostic efficacies of DKI_MD_p25, DTI_MD_p25, DTI_AD_p25 in the anterior cingulate gyrus, DTI_MD_p25, DKI_MD_p25, DTI_RD_p25 in the amygdala, and NODDI_ISOVF_p90 were also higher than 0.8. In the comparison between the PSD group and the Non-PSD group, the DTI_MD_p25 of the amygdala achieved the highest diagnostic efficacy, with an AUC of 0.816. The diagnostic efficacies of the remaining parameters were all less than 0.8.

**Table 3:** The area under the curve (AUC) in the prediction of healthy controls (HC) and post-stroke depression (PSD) * *P*<0.05 ** *P*<0.01

	AUC	95%CI	P-value
Precuneus_R_NODDI_ODI_p10	0.817	0.645 ~ 0.989	0.006**
Cingulum_Ant_L_NODDI_ODI_p10	0.806	0.626 ~ 0.985	0.008**
Cingulum_Ant_R_NODDI_ODI_p10	0.806	0.629 ~ 0.982	0.008**
Amygdala_R_NODDI_ISOVF_p90	0.806	0.641 ~ 0.970	0.008**
Amygdala_R_DKI_MD_p50	0.789	0.601 ~ 0.976	0.013*
Frontal_Mid_R_DKI_MD_p50	0.783	0.612 ~ 0.954	0.014*
Amygdala_R_DKI_MD_p75	0.772	0.600 ~ 0.945	0.019*
Amygdala_R_DKI_MD_p90	0.772	0.590 ~ 0.954	0.019*
Amygdala_R_DTI_MD_p90	0.772	0.592 ~ 0.953	0.019*
Frontal_Mid_R_DTI_RD_p50	0.767	0.575 ~ 0.959	0.021*
Amygdala_R_DTI_AD_p90	0.767	0.581 ~ 0.952	0.021*
Frontal_Mid_R_DTI_MD_p50	0.756	0.561 ~ 0.950	0.027*

**Table 4:** The area under the curve (AUC) in the prediction of healthy controls (HC) and non-post-stroke depression (Non_PSD) patients * *P*<0.05 ** *P*<0.01

	AUC	95%CI	P-value
Cingulum_Ant_L_DKI_MD_p10	0.898	0.790 ~ 1.005	0.000**
Cingulum_Ant_L_DKI_MD_p25	0.886	0.769 ~ 1.003	0.000**
Cingulum_Ant_L_DTI_MD_p25	0.874	0.752 ~ 0.997	0.000**
Amygdala_R_DKI_MD_p25	0.865	0.739 ~ 0.992	0.000**
Frontal_Mid_L_DKI_MD_p25	0.857	0.729 ~ 0.985	0.000**
Cingulum_Ant_L_DTI_AD_p25	0.854	0.723 ~ 0.984	0.000**
Frontal_Mid_R _DKI_MD_p25	0.851	0.724 ~ 0.977	0.000**
Frontal_Mid_R _DKI_MD_p25	0.845	0.711 ~ 0.979	0.000**
Amygdala_L_DKI_MD_p25	0.845	0.713 ~ 0.977	0.000**
Amygdala_L_DKI_MD_p25	0.845	0.714 ~ 0.976	0.000**
Amygdala_L_DKI_MD_p25	0.842	0.710 ~ 0.974	0.000**
Amygdala_R_NODDI_ISOVF_p90	0.81	0.659 ~ 0.961	0.001**
Cingulum_Post_R_NODDI_ISOVF_p75	0.804	0.650 ~ 0.958	0.002**

**Table 5:** The area under the curve (AUC) in the prediction of healthy controls (HC) and non-post-stroke depression (Non_PSD) patients * *P*<0.05 ** *P*<0.01

	AUC	95%CI	P-value
PSD v.s. non-PSD			
Amygdala_L_DTI_MD_p25	0.816	0.661 ~ 0.970	0.006**
Amygdala_L_DTI_AD_p25	0.795	0.633 ~ 0.957	0.010*
Cingulum_Post_R_DTI_AD_p25	0.768	0.594 ~ 0.943	0.019*
Amygdala_L_DTI_RD_p25	0.768	0.598 ~ 0.939	0.019*
Cingulum_Post_L_DKI_MD_p10	0.763	0.582 ~ 0.944	0.022*
Cingulum_Post_R_DKI_MD_p10	0.758	0.581 ~ 0.935	0.025*
Amygdala_L_DKI_MD_p10	0.758	0.584 ~ 0.932	0.025*
Amygdala_R_DKI_MD_p10	0.758	0.583 ~ 0.933	0.025*

## Discussion

This study compared three different diffusion models to evaluate their diagnostic efficacy for gray-matter microstructural changes in PSD patients, which is beneficial for further clarifying the pathogenesis of PSD and exploring potential diagnostic methods.

The main conclusions of this study are as follows: 1. Compared with the normal group, stroke patients exhibited significant microstructural changes in regions such as the precuneus, anterior cingulate gyrus, and amygdala. The amygdala showed significant differences between post-stroke depression patients and post-stroke non-depression patients. 2. For different group comparisons, different diffusion models demonstrated varying diagnostic efficacies. For the normal group and PSD patients, NODDI had the highest diagnostic value. For the normal group and post-stroke non-depression patients, both DKI/DTI and NODDI showed differences. For post-stroke depression and post-stroke non-depression patients, only DTI showed significant differences.

This study found that compared with the normal group, stroke patients had significant microstructural changes in regions such as the precuneus, anterior cingulate gyrus, and amygdala. These brain regions play crucial roles in emotion regulation and cognitive function. The precuneus is involved in functions such as self-awareness, attention, and memory. Its microstructural changes may affect information processing and emotional-cognitive integration [8]. The anterior cingulate gyrus is important for emotion regulation, cognitive control, and pain perception. Its damage may disrupt the emotion-regulation circuit [15]. The amygdala is the core region for emotion processing, associated with fear, anxiety, and emotional memory. Microstructural abnormalities may lead to emotional response disorders [16]. Some studies also support the importance of these brain regions in post-stroke functional changes. For example, Liang et al.'s study detected microstructural changes in the white matter of the frontal lobe, temporal lobe, and genu of the corpus callosum in PSD patients [17]. Although the brain regions are not exactly the same as those in this study, they all involve frontal-lobe-related regions, indicating the key role of the frontal lobe in post-stroke emotional and cognitive dysfunction. This study focused more on specific regions such as the precuneus, anterior cingulate gyrus, and amygdala, further refining the potential key brain regions.

The amygdala showed significant differences between post-stroke depression and non-depression patients, highlighting its central role in the pathogenesis of PSD. The amygdala has extensive neural connections with other brain regions and serves as a crucial hub in emotion processing and stress response. Its structural and functional changes may impact neurotransmitter transmission, neural plasticity, and neural-circuit function, thereby triggering depressive symptoms [18]. Consistent with previous studies, many investigations have found structural and functional abnormalities in the amygdala of depression patients. For instance, in some studies on general depression, changes in amygdala volume, metabolic abnormalities, and abnormal functional connections with other brain regions have been observed [19]. However, this study focused on post-stroke depression patients, indicating the unique role of the amygdala in the occurrence and development of depression in the specific pathological context of stroke, further enriching the research in this field.

For the normal group and PSD patients, NODDI had the highest diagnostic value. This is attributed to its ability to quantitatively analyze the orientation dispersion and density information of neurites in brain tissue at the micro-level. In the brain regions of PSD patients, such as the precuneus and anterior cingulate gyrus, the microstructural changes of neurites may be more complex. NODDI parameters (such as ODI, ICVF, etc.) can more accurately capture these subtle changes, thus effectively differentiating between the normal group and PSD patients [20].

In the comparison between the normal group and post-stroke non-depression patients, both DKI/DTI and NODDI showed differences. DKI can overcome the limitations of DTI and provide more detailed information about the diffusion characteristics of neural tissues by quantifying the average and directional kurtosis values and diffusion coefficients. DTI evaluates the integrity of brain white-matter fibers based on the diffusion characteristics of water molecules. NODDI supplements information from the perspective of neurite micro-structure. The three models have their respective advantages in detecting brain microstructural changes and jointly reflect that even stroke patients without depressive symptoms have certain brain microstructural changes, and different models can detect them from different dimensions [10].

For post-stroke depression and post-stroke non-depression patients, only DTI showed significant differences. This may be because the integrity of brain white-matter fibers reflected by DTI differed more obviously between the two groups of patients, and this difference was not prominently reflected in the parameters of other models. In some brain regions, such as the amygdala, the white-matter fibers of PSD patients may be more severely damaged due to stroke and subsequent pathological processes, resulting in changes in the diffusion characteristics of water molecules, which can be sensitively detected by DTI [21].

This study has several limitations that merit consideration. First, the sample size was relatively small, consisting of only 29 stroke patients and 18 healthy controls. A larger sample would enhance the statistical power, enabling more robust detection of subtle differences among groups and improving the generalizability of the findings. This small sample size may have led to less reliable results, and the conclusions drawn may not be applicable to the broader population of stroke patients.

Second, the research was restricted to acute cerebral infarction patients, excluding those with other stroke types like hemorrhagic strokes. Given that different stroke etiologies can result in distinct patterns of brain damage and subsequent PSD development, the diagnostic performance of the diffusion models may vary. As such, the current findings cannot be simply extended to all stroke patients.

Third, the cross-sectional nature of this study limits our ability to establish causal relationships between the observed microstructural changes and the development of PSD. Longitudinal studies are essential to track the temporal evolution of brain microstructural changes in relation to the onset and progression of PSD over time. This would help clarify whether the microstructural abnormalities precede the development of PSD or are a consequence of it.

Finally, the imaging analysis primarily relied on a few selected diffusion models, and other emerging diffusion-based techniques were not explored. Future research could incorporate more advanced imaging modalities, such as high-angular-resolution diffusion imaging or diffusion-weighted spectroscopy, to provide a more comprehensive understanding of the complex microstructural alterations in PSD patients. Additionally, the study did not account for the potential confounding effects of genetic factors, comorbidities, and different treatment regimens on brain microstructures and PSD. These factors may significantly influence the diagnostic performance of the diffusion models and should be considered in future investigations.

## Conclusions

In conclusion, the gray matter microstructural damage of the precuneus, cingulate gyrus, and amygdala may be involved in the pathogenesis of PSD, and the degree of damage is likely related to the severity of PSD. Multiple parameters of the NODDI model can effectively distinguish between the normal group and PSD patients. The DKI and DTI models also demonstrate the ability to diagnose the normal group and non-PSD patients and differentiate between PSD and non-PSD patients in multiple gray matter brain regions. However, different diffusion models have their own characteristics and are suitable for different clinical scenarios. Future research with larger sample sizes, inclusion of various stroke types, longitudinal designs, and exploration of advanced imaging techniques is needed to further validate the diagnostic efficacies of these models and provide more accurate and comprehensive diagnostic tools for PSD.
